# Comparison of minimally invasive and open TLIF outcomes with more than seven years of follow-up

**DOI:** 10.1016/j.xnsj.2022.100131

**Published:** 2022-06-11

**Authors:** Jae-Young Hong, Won Seok Kim, Jiwon Park, Chi Heon Kim, Hae-Dong Jang

**Affiliations:** a Department of Orthopedics, Korea University Ansan Hospital, Ansan, South Korea; b Seoul National University College of Medicine and Hospital, Seoul, South Korea; c Department of Orthopedics, Soonchunhyang University Hospital, Buchunl, South Korea

**Keywords:** Transforaminal lumbar interbody fusion, Minimally invasive, Adjacent segment disease

## Abstract

**Background:**

Few studies directly comparing minimally invasive (MI) transforaminal lumbar interbody fusion (TLIF) and open TLIF offering long-term follow-up data have been performed to date. Therefore, we sought to compare mid- to long-term outcomes between these two surgical approaches.

**Methods:**

This was a retrospective data analysis of two surgical groups. We analyzed the details of 97 patients with degenerative lumbar disease who were treated with MI TLIF (n = 55) or open TLIF (n = 42) between 2011–2014 and had at least seven years of follow-up data available. Peri- and postoperative outcomes were compared. To evaluate rates of adjacent segment disease (ASD) and revisions, frequencies of radiologic, symptomatic, and operative ASD were analyzed accordingly.

**Results:**

In terms of clinical outcome, the Oswestry Disability Index and visual analog scale scores were significantly reduced, with no difference between the groups. However, data for several peri- and postoperative outcomes, including perioperative blood loss, ambulation day, hospital stay, and operation time, varied in a manner favoring the MI TLIF group (*P* < 0.05). Rates of radiologic ASD and symptomatic ASD were significantly higher in the open TLIF group beginning at five years of follow-up (*P* < 0.05), while the rate of operative ASD and the revision rate were similar between the groups. Other long-term outcomes, including fusion rate and complications, remained similar between the two groups at 7 years.

**Conclusion:**

Patients undergoing MI TLIF showed favorable immediate postoperative outcomes and less radiographic ASD. However, the rates of fusion and operative ASD remained similar between the two groups after 7 years of follow-up.

## Introduction

Transforaminal lumbar interbody fusion (TLIF) has persisted as a good treatment option for degenerative spine diseases for several decades. Conventional TLIF has achieved good results but requires the creation of a midline incision that violates the back muscles for appropriate exposure.([1], [2], [3])

Some studies have suggested the advantages of adopting a minimally invasive (MI) spinal approach, such as less soft-tissue injury and blood loss, a reduced length of hospital stay, and decreased postoperative pain levels. In particular, the use of MI techniques for TLIF employing percutaneous pedicle screws has been reported recently to lead to less adjacent segment soft tissue injury.([4], [5], [6]) This means that the MI approach might preserve the proximal and facet complexes as well as the midline ligament complex, which can prohibit further development of adjacent segment disease (ASD). Comparisons of the two techniques have been reported, including a meta-analysis.(7,8) However, few studies directly comparing these two surgical approaches offering long-term follow-up have been conducted to date, and a direct long-term comparison study to confirm the efficacy of MI or the conventional technique might be beneficial to physicians.([9], [10], [11])

The aim of this study was therefore to compare the mid- to long-term clinical outcomes of MI and open TLIF and assess the incidence rates of ASD and revision surgery.

## Methods

### Subjects

We reviewed the medical records and radiographs of patients with lumbar degenerative diseases who underwent MI or open TLIF between 2011–2014 with ≥7 years of follow-up data (open TLIF, 2011–2012; MI TLIF, 2013–2014). We excluded patients who presented with fracture, a history of metastasis, or infection. Patients who received medicine for rheumatoid arthritis or ankylosing spondylitis with significant bony changes were also excluded.

Initially, 118 subjects were included in the study, and 21 (MI = 12; open = 9) subjects (17.8%) were excluded due to follow-up loss at any point. Thus, a total of 97 consecutive patients (MI = 55; open = 42) finally was included in this study. All operations were performed by an experienced surgeon (J. Y. H.) at our institution to minimize any variations in the learning curve and indication for surgery. We performed a power analysis to confirm the appropriate size of each group for statistical significance (effect size/power = 0.8/0.95; sample size per group = 35 participants).

In the MI TLIF group, the average patient age was 59.2 ± 5.9 years, and 28 men and 27 women were included; in the open TLIF group, the average patient age was 56.7 ± 4.7 years, and 20 men and 22 women were included (Table 1). Among these patients, 55 underwent single-level TLIF procedures (MI = 25; open = 30) and 42 underwent two-level TLIF procedures (MI = 25; open = 17). Regarding the level of operation, 79 cases involved L4/L5, 49 cases involved L5/S1, and 11 cases involved L3/L4. All patients underwent pre- and postoperative evaluations with neurologic examination and radiologic imaging. Patients were followed up regularly after surgery (i.e., at two weeks, three and six months, and one year, and annually thereafter), and postoperative symptoms and complications were observed at every time point. An institutional review board approved the study (approval no. AS0133), and the need to gather patient informed consent was waived.

**Table 1:** Comparison of demographics between the two study groups

Comparison of demographics between the two study groups			
	MI TLIF	Open TLIF	P-value
**Number of subjects**	55	42	
**Sex (M:F)**	28:27	20:22	0.104
**Age (years)**	59.2 ± 5.9	56.7 ± 4.7	0.190
**BMI (kg/m^2^)**	25.7 ± 2.1	25.1 ± 3.0	0.177
**Smoking**	15	9	0.146
**Diabetes**	7	5	0.187
**Hypertension**	12	11	0.102
**Renal disease**	4	2	0.166
**Mean follow-up years**	7.7 ± 1.2	7.2 ± 1.9	0.091
**Diagnosis**			
**Spinal stenosis** **Spondylolisthesis** **HIVD** **DDD** **Segmental instability**	36 10 2 3 4	30 7 1 2 2	0.083
**Disease Level**			
**L3/4**	6	5	0.112
**L4/5**	43	36	
**L5/S1**	31	18	

BMI: Body mass index; HIVD: herniated intervertebral disc; DDD: degenerative disc disease

Diabetes, hypertension, and renal disease were confirmed as when the patient was regularly taking medicine for their condition.

The t-test and chi-squared test were used to determine differences between the two groups.

No significant differences existed between the two groups (P > 0.05).

### Surgical technique

#### MI TLIF approach and percutaneous screw fixation

**Figure 1:**
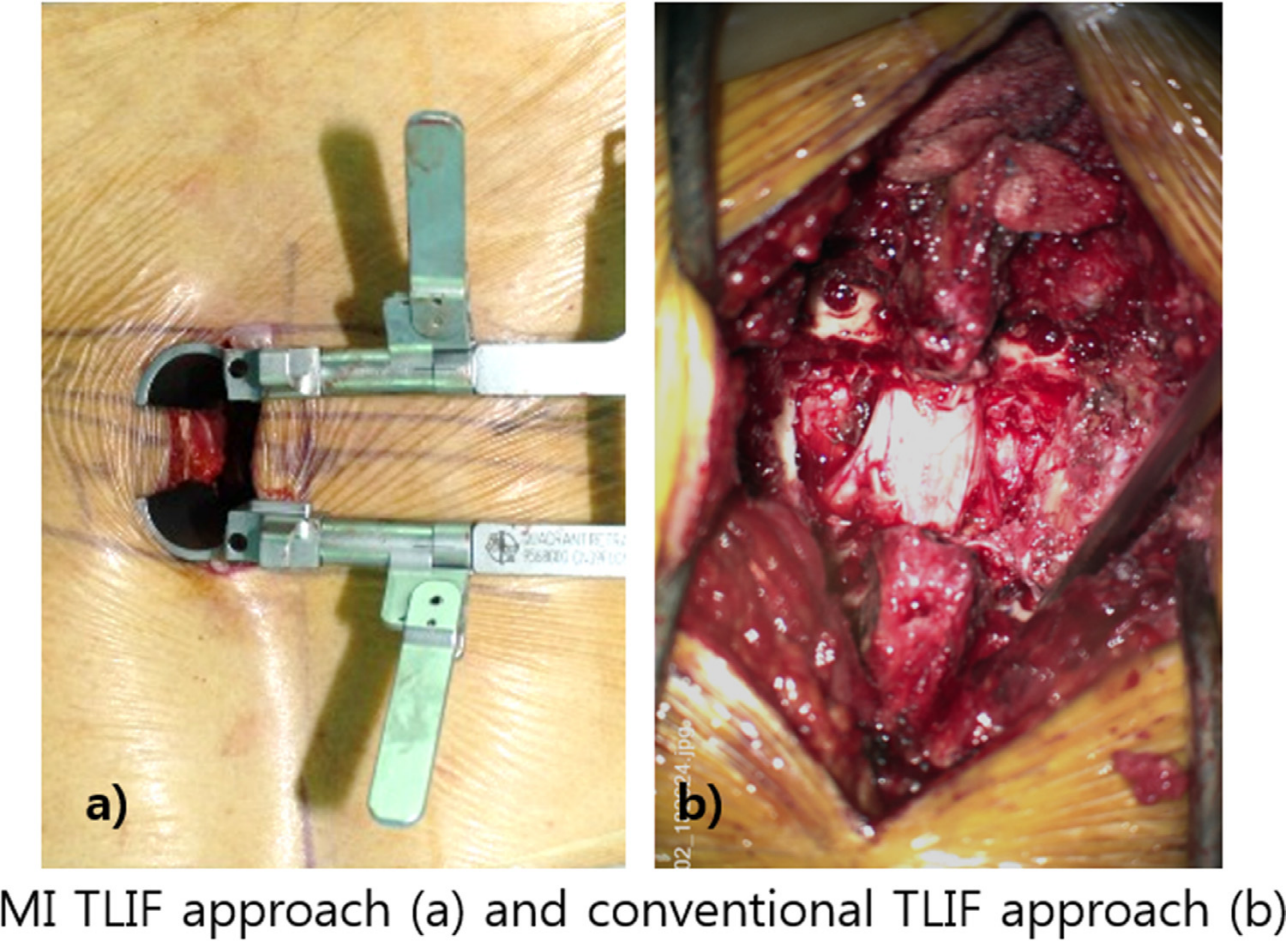
MI TLIF approach (a) and conventional TLIF approach (b).

During this procedure, a lateral skin incision was created on the symptomatic side. The muscle fascia was incised in line with the skin incision, and the muscle plane between the multifidus and longissimus muscles was identified with a gloved finger. Dilating instrument retractors (METRx; Medtronic, Minneapolis, MN, USA) were attached, and a decompression maneuver was completed. Inter-body fusion with cage was performed with harvested lamina and facet bone and demineralized bone matrix (Grafton; Medtronic, Minneapolis, MN, USA). For percutaneous pedicle screw fixation, a contralateral skin incision was made through the intermuscular plane, bilateral pedicle screws were placed on the corresponding level, and a rod was inserted percutaneously under fluoroscopic guidance (Longitude; Medtronic, Minneapolis, MN, USA) (Fig. 1a).

### Open TLIF approach and conventional screw fixation

In contrast, during this procedure, a midline skin incision was first created, and the muscle fascia was incised in line with the skin incision; then, the spinous process and interspinous ligament complex were identified. Meticulous dissection was performed through the proximal facet to distal facet joint with a Bovie dissector, and decompression was completed, including removal of the inter-spinous ligament complex. Inter-body fusion with cage was performed with harvested lamina and facet bone and demineralized bone matrix (Grafton; Medtronic, Minneapolis, MN, USA). Bilateral pedicle screws were placed at the anatomical position on the corresponding level manually, and correct screw position was confirmed with fluoroscopy (Fig. 1b).

#### Outcomes assessment

A mean visual analog scale (VAS) for leg pain and the Oswestry Disability Index (ODI) were used to assess pain and disability. For radiologic outcome parameters, both static and dynamic plain lumbar radiography and computed tomography were performed at six months and two years, respectively, according to the Bridwell grading system.(12)

For perioperative parameters, operation time, blood loss, length of hospital stay, and day of postoperative ambulation were assessed to evaluate clinical outcomes. Cases of major complications were also recorded in both groups, and rates of radiographic ASD (R-ASD), symptomatic ASD (S-ASD), and operative ASD (O-ASD) were evaluated. Data were reviewed annually beginning at one year after surgery until the final follow-up date. R-ASD was defined as radiographic degeneration adjacent to the fused segment on plain radiographs, including narrowing of disc height (>3 mm), anterior or posterior listhesis (>3 mm), and posterior opening (>5°).(13) S-ASD was defined as a symptomatic condition due to neurological deterioration of the ASD, and O-ASD was defined as a revision surgery performed at an adjacent segment. All radiographic parameters were evaluated by two independent observers (J. Y. H. and W. S. K.).

### Statistical analysis

For comparisons between the two groups, the *t*-test and chi-square test were used to compare each parameter. The Statistical Package for the Social Sciences version 20.0 software program (IBM Corporation, Armonk, NY, USA) was used for the analyses.

## Results

### General clinical outcomes

The mean ODI scores of the two groups were significantly decreased postoperatively (*P* < 0.05), with no significant difference between them, and were maintained (*P* > 0.05). In addition, the mean VAS score was reduced from 5.7 ± 2.0 to 2.8 ± 1.1 points postoperatively, also with no significant difference present between the two groups (*P* > 0.05). The mean postoperative changes in VAS and ODI scores were similar between the two groups (*P* > 0.05).

The mean operative time for the MI TLIF group was 188.2 ± 42.2 min (per one level), and the mean operative time for the open TLIF group was 209.6 ± 26.9 min (*P* < 0.05). The mean amounts of blood loss during MI and open TLIF were 246.2 ± 45.8 and 299.1 ± 22.4 mL, respectively, and the mean total amounts of drainage after surgery were 194.1 ± 20.7 and 402.2 ± 33.4 mL, revealing significant differences between the two groups (*P* < 0.05).

The transfusion rate was different between two groups in favor of MI group (*P* < 0.05). The mean numbers of days to ambulation for patients who underwent MI and open TLIF, respectively, were 1.1 ± 0.2 and 1.9 ± 0.4 days (*P* < 0.05), and the lengths of hospital stay for the same groups were 5.1 ± 0.4 and 7.9 ± 0.3 days (*P* < 0.05).

There were three instances of major complications after surgery in the MI group, including one case of postoperative infection and two cases of screw loosening and nonunion; each of these cases was treated conservatively. In the open group, there were four major complications: one patient experienced postoperative infection; one patient experienced screw loosening and nonunion; and two patients experienced incomplete root damage, which was resolved by three months. All of these complications were also treated without surgery (Table 2).

**Table 2:** Comparison of pre- and postoperative outcomes

Comparison of pre- and postoperative outcomes				
Outcome & Complication				
	MI TLIF	Open TLIF	Mean	P-value
**OP time (min)**	188.2 ± 42.2	209.6 ± 26.9	200.4 ± 29.5	0.044
**Intra-OP drain (mL)**	246.2 ± 45.8	299.1 ± 22.4	274.2 ± 30.1	0.034
**Post-OP drain (mL)**	194.1 ± 20.7	402.2 ± 33.4	301.5 ± 34.1	0.002
**Total blood loss (mL)**	411.6 ± 25.5	659.7 ± 21.0	551.8 ± 31.0	0.010
**Transfusion (%)**	3.6%	11.9%	7.2%	0.018
**Ambulation day**	1.1 ± 0.2	1.9 ± 0.4	1.4 ± 0.3	0.021
**Hospital stay**	5.1 ± 0.4	7.9 ± 0.3	6.8 ± 0.3	0.045
**Complications**	1 infection	1 infection		0.155
	1 non-union	1 non-union		
		2 N.damage		
**Pre-VAS** **Pre-ODI**	5.5 ± 1.1 25.1 ± 2.4	5.8 ± 0.9 27.0 ± 3.1	5.7 ± 2.0 25.5 ± 1.0	0.124 0.177
**Post-VAS** **Post-ODI**	2.8 ± 0.3 15.7 ± 3.1	2.8 ± 1.0 16.0 ± 1.7	2.8 ± 1.1 15.9 ± 1.1	0.101 0.099
**Post-OP change-VAS**	2.5 ± 1.3	2.7 ± 0.8	2.7 ± 1.2	0.141
**Post-OP change-ODI**	11.5 ± 3.3	12.5 ± 2.1	12.1 ± 2.3	0.091

**ODI**: Oswestry Disability Index; **OP**: operative; **VAS**: visual analog scale; **N**: nerve.

The **t-test** and **chi-square test** were used to determine differences between the two groups.

### Union status and ASD

According to Bridwell's classification, we divided the union status into four grades (Table 3). We found that 85.5% of patients in the MI TLIF and 90.5% in the open TLIF showed satisfactory union status (grades I and II), with no significant intergroup difference.

**Table 3:** Union status and ASD

Union status and ASD			
	MI TLIF (n = 55)	Open TLIF (n = 42)	P-value
**I & II**	47 (85.5%)	38 (90.5%)	0.345
**III**	6 (10.9%)	3 (7.1%)	
**IV**	2 (3.6%)	1 (2.4%)	
**ASD Yrs**	**R/S/O**	**R/S/O**	**R/S/O**
**1**	1/0/0	1/0/0	0.102/NA/NA
**2**	3/2/0	3/3/0	0.119/0.125/NA
**5**	12/8/4	15/12/5	0.023/0.019/0.466
**7**	13/9/4	17/13/5	0.019/0.022/0.199

**ASD**: adjacent segment disease; **O**: operative adjacent segment disease; **R**: radiologic adjacent segment disease**; S**: symptomatic adjacent segment disease**; NA**: not applicable.

**Bridwell's classification** was used.

Grade I: fused with remodeling and trabeculae present; grade II: graft intact, not fully remodeled and incorporated, but no lucency present; grade III: graft intact, potential lucency present at top and bottom of graft; and grade IV: fusion absent with collapse/resorption of graft.

The **t-test** and **chi-square test** were used to determine differences between the two groups.

**Figure 2:**
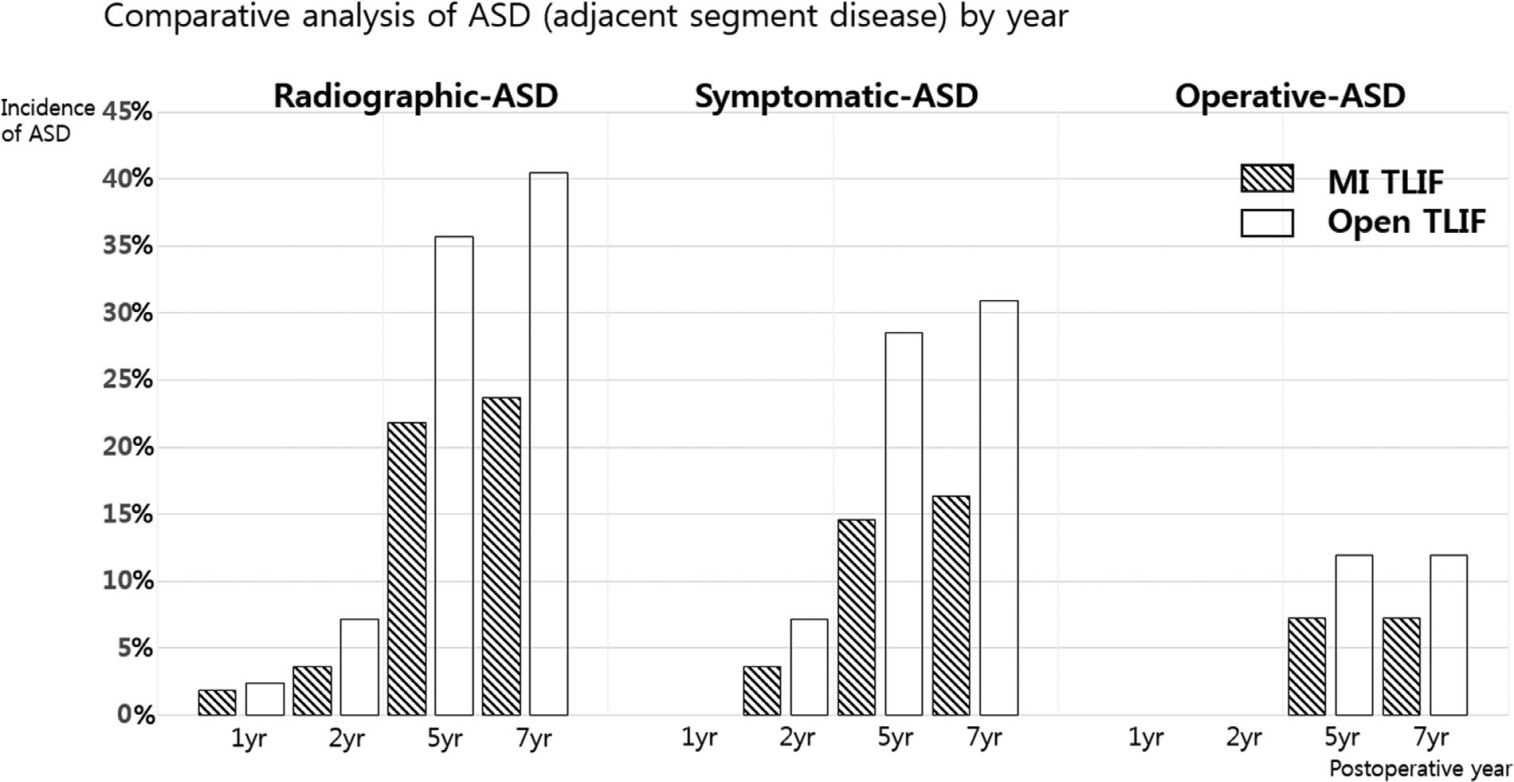
Comparative analysis of ASD by year.

ASD parameters were increased every year in both groups and were significantly different after five years (*P* < 0.05). R-ASD was found in 12 cases in the MI group and 15 cases in the open group (21.8% vs. 35.7%) at five years of follow-up; as such, the open group showed a significantly greater incidence of R-ASD than the MI group (*P* = 0.023). Incidence rates of S-ASD also differed between the MI and open TLIF groups (14.5% vs. 28.6%) at five years of follow-up, increasing until final follow-up, while those of O-ASD were not different between the groups at seven years (7.2% vs. 11.9%) (Fig. 2).

## Discussion

Conventional TLIF is a technique based on the traditional posterior lumbar interbody fusion technique. Its advantages include reductions in the incidence rates of traction and direct injury of the nerve root and dural sac and preservation of bony structures as well as ligaments and muscles on the midline and contralateral side.([1], [2], [3]) Surgeons have sought to adopt various MI surgical techniques to treat lumbar degenerative diseases, which can lessen the degree of soft tissue injury. These efforts began with mini-open or laparoscopic anterior lumbar fusion and have progressed to the current MI posterior lumbar fusion technique.(5,6,[9], [10], [11], [12], [13], [14], [15], [16], [17], [18], [19], [20])

With the advancements in spinal instrumentation and radiologic imaging, MI TLIF represents an alternative to conventional TLIF. Foley et al. described this technique with percutaneous pedicle screws, which rely on tubular retractors inserted serially under radiologic guidance by a muscle-dilating approach, thus reducing damage to the iatrogenic muscle and soft tissues.(5) This technique can be considered another evolution of the classical open TLIF method given patients’ fast recovery and reduced length of hospital stay after surgery as well as more limited blood loss.

Surgeons remain concerned about incomplete decompression or significant delay of the operation time with an MI approach, which can induce poor clinical outcomes. In our study, the average ODI and VAS scores after MI were significantly reduced and remained as such throughout the follow-up period, which did not differ from the clinical results obtained using the open TLIF method. Similar clinical efficacies of MI TLIF have been reported in numerous studies comparing this method with conventional TLIF.([21], [22], [23], [24]) In addition, we confirmed the achievements of less blood loss and faster recovery times, which can be the most critical findings attributed to MI surgery.

In our study, the mean postoperative time to ambulation was 1.1 ± 0.2 days, and the mean amount of blood loss during surgery was 246.2 ± 45.8 mL, which confirms that MI TLIF culminates in less intraoperative blood loss than conventional TLIF. The drainage volumes after surgery were significantly different (194.1 vs. 402.2 mL), which might be related to a reduced dead space after MI relative to the open approach. The difference in transfusion rate between the two groups (3.6% vs. 11.9%) also supports our hypothesis. In addition, MI TLIF significantly reduces damage to posterior structures, including muscles and ligaments, which enables patients to walk within a few days after surgery. Early ambulation enhanced the recovery of patients after surgery, which can minimize postoperative morbidity. Similarly, Chan et al. confirmed a 2.5-fold reduction in the amount of intraoperative blood loss compared to that seen during open TLIF.(25) A significantly reduced degree of blood loss has also been reported in other MI TLIF series, thus reducing the need for blood transfusions and associated risks.

Although operative time was significantly different between our two groups, the difference was very small, and we could not confirm which method was better. However, regardless of the difficulty of the technique and stiff learning curve, we could not confirm a significant difference in the rate of complications between MI and conventional TLIF. We thought therefore that MI TILIF might be effective in improving the functional ability of patients with degenerative lumbar disease and could be more efficient with an increase in the surgeon's familiarity with the technique.(26,27)

The achievement of solid fusion after TLIF has been a significant issue inherent with MI approaches due to the lack of sufficient grafting of local bone. In this study, 85.5% of patients achieved solid fusion in the MI TLIF group, which was not different from the union rate noted in the conventional group. There are many reports documenting similar rates of successful fusion between MI and open TLIF.(15,16,19,25) We thought that preserving the midline ligament–bone complex would provide additional stability that is favorable for solid fusion regardless of the amount of bone graft. However, a greater increase in the solid fusion rate might minimize costs related to pseudoarthrosis in the MIS group.

ASD or degeneration remains one of the most concerning long-term complications and can cause recurrent issues following spinal fusion for spinal disease. Ghiselli et al. reported that the rate of ASD was 16.5% at five years and 36.1% at 10 years after lumbar fusion.(28) Zhong et al. reported similar numbers, with an overall incidence rate of ASD of 11.7%, in patients undergoing fusion for spondylolisthesis.(29) In terms of the risk factors for ASD after lumbar spinal fusion, age, sex, obesity, pre-existing degeneration, number of fused levels, and type of fusion can influence the onset of ASD.

In our study, the overall surgery rate for ASD was 9.3% after MI and open TLIF, with a mean follow-up length of seven years. Generally, all ASD parameters were similar between the two groups until two years of follow-up. However, the R-ASD and S-ASD rates grew significantly greater in the open TLIF group beginning at five years of follow-up, while the rates of O-ASD and revision remained similar in the two groups. Thus, our results are consistent with those of previous reports, most of which adopted shorter follow-up times than that of our study.(25,29) This means that the MI approach can preserve the proximal and distal facet complexes as well as the midline ligament complex, which could prohibit further development of ASD. However, there are many cases of ASD that appear without or with only mild symptoms, and only severe degrees of ASD might require surgical treatment. The occurrence of R-ASD does not necessarily require prompt operative treatment. Physicians should consider clinical symptoms as well as the severity of R-ASD. Also, patients’ decision to be operated on or not might influence the re-operation rate. In addition, other outcomes, including fusion rate and complications, remained similar between our two groups during seven years of follow-up, which may have diluted the efficacy of the MI technique.

There are several limitations to our study. First, our investigation was not a randomized or a prospective study, and we did not blind the observers measuring the outcomes in the two groups. A retrospective analysis of a single institution carries a significant selection bias, which might be the critical limitation of our study. Although the indications for MI or open TLIF were not different according to our medical records, the statistical significance is far lower than that of a randomized study.

Second, there are numerous factors that affect the operative parameters. ASD can be affected by pelvic incidence or lumbar lordosis, which were not considered in this study. Different levels and fused segments, age, and pre-existing degeneration can affect the incidence of ASD but were not analyzed in this study. The severity of R-ASD can be an important factor, but we could not use the grading system in this study. Narcotics usage, which we could not analyze in this study, might influence perioperative parameters.

Moreover, there were many factors not compared in this study or that were underpowered due to a low incidence of events (e.g., complications, non-union). Lasty, regardless of the advantages of MI TLIF, it is surgically demanding and challenging for beginners to perform; difficulties in mastering this technique arise due to a limited view of the surgical field and the greater surgical finesse required with longer surgical instruments, which can limit the efficacy of MI TLIF. Inadequate decompression might also have been an issue for MI TLIF, especially for beginners. However, we could not evaluate postoperative MRI or leg-specific VAS scores to check complete decompression, which was a limitation of this study.

## Conclusion

Patients undergoing MI TLIF presented favorable immediate postoperative outcomes and less radiographic ASD than those in the open TLIF group. However, the fusion rate and operative ASD rate were similar between the two groups after seven years of follow-up, which might decrease the clinical significance of the MI technique.

## Declaration of Competing Interest

The authors declare that they have no known competing financial interests or personal relationships that could have appeared to influence the work reported in this paper.

## Appendix Supplementary materials

Image, application 1

Image, application 2

Image, application 3

Image, application 4

Image, application 5

Image, application 6
