# Supplementary material for: Comparison of minimally invasive and open TLIF outcomes with more than seven years of follow-up
Source: N Am Spine Soc J. 2022 Jun 11;11:100131. doi: 10.1016/j.xnsj.2022.100131 (PMC9243253; doi:10.1016/j.xnsj.2022.100131)
Supplement: Supplementary file 6 [file mmc6.pdf]

// THE SPINE JOURNAL
A Multidisciplinary Journal of Spinal Disorders
Official Journal of the North American Spine Society

FDA APPROVAL STATUS

Article Title: A comparative assessment of outcomes following minimally invasive versus conventional transforaminal lumbar interb

Manuscript reference #:

FDA Approval Status

If a device or drug requiring FDA approval is identified as an important component of your article, you must indicate the FDA status for use as it will be discussed. Please list the name of the device(s) and drug(s) requiring FDA approval and check the appropriate status for use as it is discussed in the article.

☒ My manuscript does not discuss any drugs or devices requiring FDA approval.

1. Device/Drug

(●) Not Applicable ( ) Not approved for this indication ( ) Approved ( ) Investigational

2. Device/Drug

(●) Not Applicable ( ) Not approved for this indication ( ) Approved ( ) Investigational

3. Device/Drug

(●) Not Applicable ( ) Not approved for this indication ( ) Approved ( ) Investigational

4. Device/Drug

(●) Not Applicable ( ) Not approved for this indication ( ) Approved ( ) Investigational

5. Device/Drug

(●) Not Applicable ( ) Not approved for this indication ( ) Approved ( ) Investigational

Corresponding Author Name: Jae-Young Hong

Date: Dec-13-2021
